# 

**Published:** 1894-12-22

**Authors:** 


					^ PRICE TWOPENCE. I1U WITH EXTRA NURSING SUPPLEMENT.
No> 430, Vol. XVII.?Dec. 22nd, 1894. Ml JJnl m U *er J0s-6d- poffree-
rt? . ' ' Bk ?    Monthlv PartH. fid. ? nnat. frAA fi
IK- . . ' ? I ? JSL^ . Monthly Parts, 6d.; post free, 8d.
Watered at the General Post Office as a Newspaper J ^ Ditt0 per Anmun> 8s. 6d> post fcee#
LAscotr Western Infirm am ^| ?NOfif.OLK*?o NORWICH HOSPI.taj.
No. 430. Vol. XVII. WITH EXTRA NURSING SUPPLEMENT. Saturday, Dec. 22nd, 1894
The CHRISTMAS NUMBER.

				

